# Trp53 Deletion Promotes Exacerbated Colitis, Facilitates Lgr5+ Cancer Stem Cell Expansion, and Fuels Tumorigenesis in AOM/DSS-Induced Colorectal Cancer

**DOI:** 10.3390/ijms252010953

**Published:** 2024-10-11

**Authors:** Anderson F. Cunha, João M. Delou, Pedro S. Barbosa, Julia S. M. Conceição, Karen C. S. Souza, Vera Chagas, Rossana C. Soletti, Heitor S. P. de Souza, Helena L. Borges

**Affiliations:** 1 Instituto de Ciências Biomédicas, Universidade Federal do Rio de Janeiro, Rio de Janeiro 21941-90, RJ, Brazil; 7andersonfaletti7@gmail.com (A.F.C.); joao.delou@idor.org (J.M.D.); phsalesbarbosa@gmail.com (P.S.B.); juliasoutomc@gmail.com (J.S.M.C.); 2 Instituto D’Or de Ensino e Pesquisa, Rio de Janeiro 22281-100, RJ, Brazil; 3 Departamento de Clínica Médica, Faculdade de Medicina, Universidade Federal do Rio de Janeiro, Rio de Janeiro 21941-913, RJ, Brazil; karenecris480@ufrj.br (K.C.S.S.); heitor.souza@gmail.com (H.S.P.d.S.); 4 Departamento de Patologia, Universidade Federal do Rio de Janeiro, Rio de Janeiro 21941-913, RJ, Brazil; veraachagas@gmail.com; 5 Departamento Interdisciplinar, Universidade Federal do Rio Grande do Sul, Tramandaí 95590-000, RS, Brazil; rossana.soletti@ufrgs.br

**Keywords:** colorectal cancer, p53, Lgr5, inflammatory bowel disease

## Abstract

Colorectal cancer CRC remains one of the leading causes of cancer-related deaths worldwide, with chronic intestinal inflammation identified as a major risk factor. Notably, the tumor suppressor *TP53* undergoes mutation at higher rates and earlier stages during human inflammation-driven colon tumorigenesis than in sporadic cases. We investigated whether deleting *Trp53* affects inflammation-induced tumor growth and the expression of Lgr5+ cancer stem cells in mice. We examined azoxymethane (AOM)/dextran sodium sulfate (DSS)-induced colon tumorigenesis in wild-type *Trp53 (+/+)*, heterozygous *(+/−)*, and knockout *(−/−)* mice. *Trp53−/−* mice showed increased sensitivity to DSS colitis and earlier accelerated tumorigenesis with 100% incidence. All groups could develop invasive tumors, but knockouts displayed the most aggressive features. Unlike wild-type CRC, knockouts selectively showed increased populations of Lgr5+ colon cancer stem-like cells. *Trp53* loss also boosted laminin, possibly facilitating the disruption of the tumor border. This study highlights how *Trp53* deletion promotes the perfect storm of inflammation and stemness, driving colon cancer progression. *Trp53* deletion dramatically shortened AOM/DSS latency and improved tumor induction efficiency, offering an excellent inflammation-driven CRC model.

## 1. Introduction

Colorectal cancer (CRC) is the third most common cancer worldwide and the second leading cause of cancer deaths, with over 1.1 million new cases and 557,000 deaths in 2020 [1]. Major risk factors are smoking, high-fat diets, age, and inflammatory bowel disease (IBD) [2,3]. IBD-associated CRC risk primarily arises from chronic inflammation, which can cause DNA damage and pre-neoplastic effects. Increased nitric oxide synthase expression promotes reactive oxygen species (ROS) formation. This oxidative stress can lead to p53 loss-of-function mutations [4].

The tumor suppressor protein p53, encoded by *TP53* in humans and *Trp53* in mice, is frequently mutated in CRC. *TP53* mutation rates increase from 15–30% in pre-malignant tumors [5,6] to 55–80% in metastatic CRC [7,8]. Notably, *TP53* mutations occur even earlier and more often (up to 89%) in IBD-related CRC [9].

p53 regulates many critical cellular processes. In the tumor microenvironment, p53 modulates crosstalk between cancer cells and immune cells, fibroblasts, extracellular matrix, and cytokine release [10,11,12]. Its loss of function can promote tumor development and progression through its effects on proliferation, cell death, angiogenesis, invasion, inflammation, and stemness [11,13,14].

Intestinal stem cells expressing Lgr5 reside at the base of intestinal crypts [15,16] and generate all epithelial lineages. A subset of mutated Lgr5+ stem cells may become cancer stem cells (CSCs), which drive tumor initiation and growth due to their self-renewal and differentiation abilities. CSCs have been implicated as responsible for tumor initiation, progression, therapy resistance, relapse, and metastasis [16,17,18,19].

While p53 is usually inactive in stem cells, its activation helps drive differentiation [20]. *Trp53* deficiency in mice impedes intestine villus aging, restraining the decline of Lgr5+ cells within crypts [21]. While the number of analyzed animals was limited, a comprehensive study revealed that *Trp53*-specific LGR5+ intestinal cell deletion augmented colorectal tumor size and incidence in the AOM/DSS model [22].

The widely used AOM/DSS mouse model induces colon rectal tumors, adenomas, and adenocarcinomas by administering azoxymethane (AOM) to initiate tumor formation followed by repeated oral administration of dextran sodium sulfate (DSS) to promote tumor progression by incorporating inflammation. The limitations are minimal invasion/metastasis and infrequent p53 mutations [23]. However, since p53 mutations occur early in human IBD-CRC [24,25,26], examining AOM/DSS tumor development in *Trp53−/−* mice could reveal valuable insights into aggressive tumor and microenvironment interactions. Here, we discuss how *Trp53* loss impacts AOM/DSS tumor growth and Lgr5 expression.

## 2. Results

**Figure 1:**
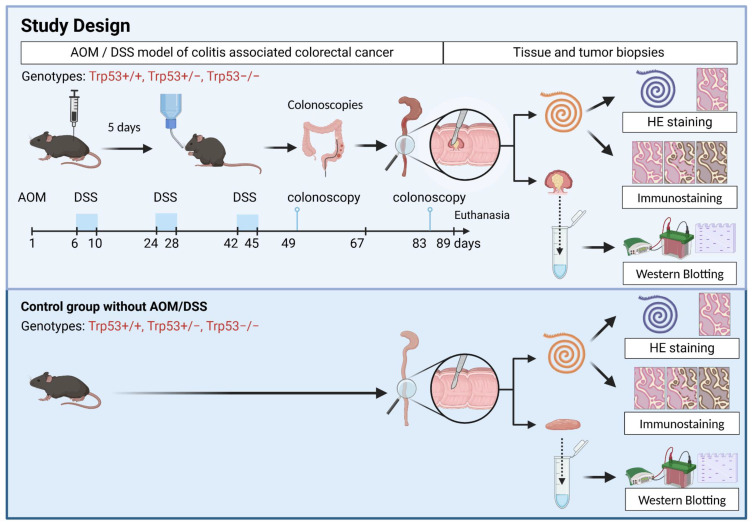
Experimental Design. The experimental design scheme of the AOM/DSS model (**upper panel**): *Trp53+/+*, *Trp53+/−*, and *Trp53−/−* mice were managed during colitis-associated colorectal induction cancer. Five days after I.P. injection of azoxymethane (AOM 12.5 mg/kg), three sodium dextran sulfate (DSS) cycles with the indicated days were performed at 2-week intervals to induce inflammation. The colonoscopy events were performed in the ninth and twelfth week to monitor tumor formation. The experimental design scheme of the control animals (**lower panel**): *Trp53+/+*, *Trp53+/−*, and *Trp53−/−* mice were used as the control group. After euthanasia, all animals have their colon and rectum removed, opened, cleaned, and biopsied. The biopsies were snap-frozen for posterior Western blotting analyses. “Swiss roll” colons were fixed and processed for paraffin embedding. Histological analysis confirmed normal tissue or tumors before biopsies were used for protein extraction.

This investigation compares control and AOM/DSS-treated colon and rectum tissues across different *Trp53* genotypes. We seek to evaluate the influence of the *Trp53* genotype in the expression of Lgr5+ stem cells in normal and dysplastic tumors. The study utilizes adult *Trp53* wild-type (*+/+*), heterozygous (*+/−*), and knockout (*−/−*) mice, both male and female, split into control and AOM/DSS treatment groups. The AOM/DSS group was around 11 weeks old (median) when they received the injection of the carcinogen AOM. Five days after AOM, animals received three cycles of the inflammatory agent DSS administered in their drinking water. Control mice received no treatment. We performed colonoscopies to monitor tumor development at weeks 9 and 12 after the beginning of the AOM/DSS protocol (Figure 1).

### 2.1. Trp53 Showed Increased Colitis

**Figure 2:**
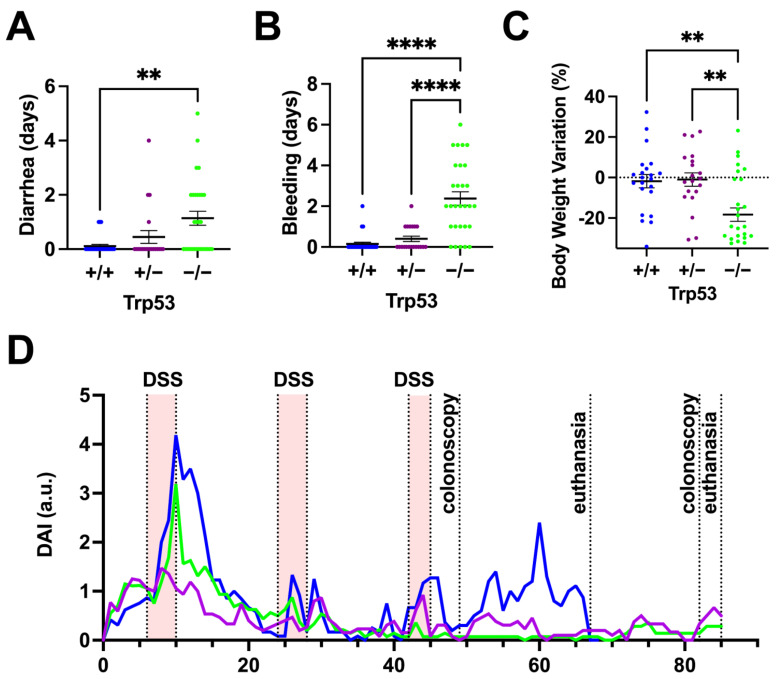
The *Trp53−/−* mice showed increased sensitivity to DSS-induced colitis. (**A**,**B**) Duration in days that colitis-associated effects like diarrhea according to the genotype and bleeding accumulated among the Trp53 genotypes during the AOM/DSS protocols *Trp53+/+*, *Trp53+/−*, and *Trp53−/−* ** *p* 0.0011, **** *p* < 0.0001, One-Way ANOVA—Tukey; (**C**) percentage of weight lost by the time of euthanasia. *Trp53+/+* vs. *Trp53−/− p* < 0.0018 and *Trp53−/−* vs. *Trp53+/− p* < 0.0014. One-Way ANOVA—Tukey; (**D**) the disease activity index (DAI) is shown according to the genotype. *Trp53−/−* vs. *Trp53+/+* and *Trp53+/−*, *p* < 0.0001.

Routine monitoring of the experimental cohort, including weighing the mice and analyzing their health status for signs of bleeding and diarrhea, is necessary to perform the AOM/DSS model. The wild-type and heterozygous mice recovered their initial weight after a temporary decline. *Trp53−/−* showed increased susceptibility to colitis symptoms, resulting in more prolonged and frequent diarrhea and bleeding events than wild-type and heterozygous mice (Figure 2A,B). Consistent with increased DSS-induced colitis, *Trp53−/−* mice had an average weight drop of 17% compared to their initial weight (Figure 2C).

The combination of diarrhea, hematochezia, and body weight forms a Disease Activity Index (DAI) analysis according to the criteria described in Table S1. Immediately after AOM injection, the *Trp53+/+* and *Trp53+/−* mice showed increased DAI compared to the *Trp53−/−* mice (Figure 2D). All DSS cycles induced DAI elevations across all their genotype groups, but the *Trp53−/−* mice had increased DAI after all DSS treatments compared to other genotypes. The *Trp53−/−* and *Trp53+/−* mice had increased DAI particularly in the first higher DSS cycle of 2%, responding better after the following two DSS cycles of 1.5%. Near day 60 of the beginning of the AOM/DSS, the *Trp53+/−* mice had an acute increase in DAI unrelated to a DSS cycle. By day 67, most of the animals’ *Trp53−/−* had reached the endpoint criteria, they were euthanized, and their intestines were dissected. The colons and rectums were opened, photographed, and biopsies of large tumors were frozen for qPCR and Western blot analysis. The intestines were then prepared as ‘Swiss rolls’ and processed for histological analysis.

During the autopsy, the *Trp53−/−* mice had their thymus macroscopic examination to exclude large thymic lymphomas as a significant cause of the increased DAI since thymic lymphomas have high penetrance in these animals [27].

In the ninth week of the AOM/DSS protocol, we performed the first colonoscopy of the animals. Colonoscopy imaging was performed in AOM/DSS-treated mice, and the severity of colorectal inflammation and tumorigenesis was scored during the procedure according to Figure S1. Due to high rates of perianal lesions in the *Trp53−/−* mice, most of the animals from this group could not be adequately evaluated by colonoscopy. Two *Trp53−/−* mice were examined, with an average inflammation score of 4, contrasting with average scores of 3.2 for *Trp53+/−* (*n* = 10) and 3.1 for *Trp53+/+* (*n* = 15) mice (Figure S2A). More importantly, *Trp53−/−* mice obtained maximal tumor scores of (2), whereas *Trp53+/−* showed 0.6 and *Trp53+/+* 0.8 in colonoscopy imaging, showing that only *Trp53−/−* showed multiple tumors in the ninth week of AOM/DSS administration (Figure S2B). Representative colonoscopy images showed a single tumor and contact bleeding in *Trp53+/+*, thickened mucosa in *Trp53+/−*, and multiple tumors and contact bleeding in *Trp53−/−* mice (Figure S2C). *Trp53+/+* and *Trp53+/−* animals were subject to predicted euthanasia at the end of the AOM/DSS experiment and had their colons and rectums removed for subsequent analysis.

### 2.2. Tumor Efficiency and Colon Analysis

**Figure 3:**
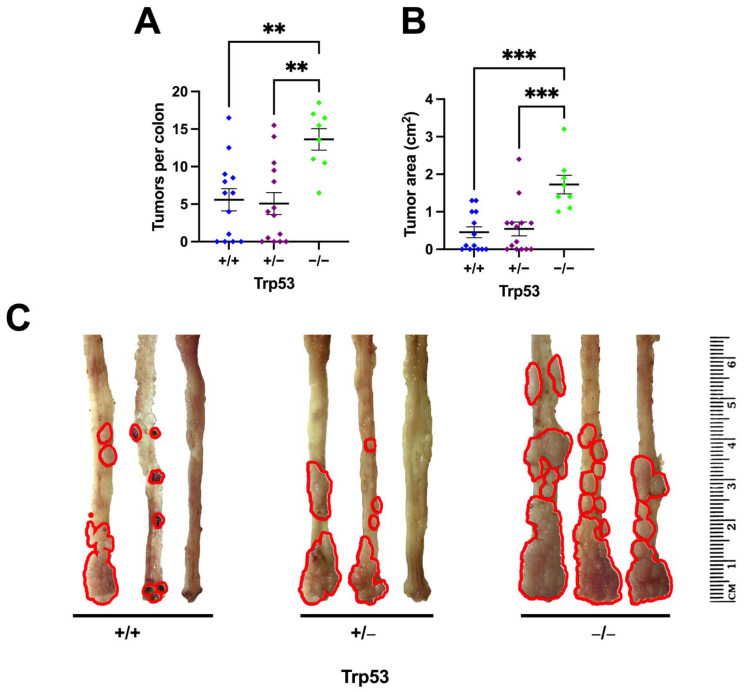
Tumor efficiency and colon analysis. (**A**) Number of tumors per colon after dissection. *Trp53−/−* showed twice as many tumors as other animals. ** *p* < 0.0020 (*+/−* vs. *−/−*), ** *p* < 0.0041 (*+/+* vs. *−/−*). One-Way ANOVA—Tukey; (**B**) tumor area per colon. Significant difference in tumor area of *Trp53−/−* (*** *p* < 0.0003) to other groups; (**C**) representative pictures of animals’ colons and rectums after euthanasia. Tumors are highlighted in red.

A general assessment of the colons was performed for four parameters after euthanasia: colon length, tumor area, tumor count, and percentage of animals with tumors. All parameters were analyzed, comparing genotypes, gender, and age. When analyzing tumor area and tumor count, we found an expected result concerning the knockout group, which showed a significant difference from the wild-type and heterozygous groups (Figure 3A,B). The average number of tumors of *Trp53−/−* mice was more than double that of the other genotypes (Figure 3A,C). 

We also compared the lengths of the large intestine after AOM/DSS treatment and found no significant differences between genotypes (Figure S3A). To investigate this further, we analyzed the entire “Swiss roll” of the colon using an inflammation scoring system (maximum score = 10) described in the Methods section. Consistent with no difference in colon length, we also did not detect differences between genotypes using this inflammation score (Figure S3B), suggesting that by the time the tumors were fully developed, and adenocarcinomas were frequently found in all genotypes (Figure S4), inflammation levels in the whole colon were comparable between genotypes (Figure S3B). We then examined immune cell infiltration specifically in adenocarcinomas and found a slight increase in infiltration in *Trp53−/−* tumors compared to *Trp53+/+* (Figure S3C). Despite this, qPCR analysis of several inflammation markers, including NF-κB, TNF-α, CD68, IL-6, IL-10, CCL2, and TGF-β, revealed no significant differences in mRNA levels between tumors from *Trp53+/+* and *Trp53−/−* mice (Figure S3D). Overall, although *Trp53−/−* mice exhibited clinical signs of over-inflammation following DSS treatment, by the time the adenocarcinomas were already present, we could not detect a strong inflammatory signal, aside from a slight increase in immune cell infiltration in *Trp53−/−* tumors.

### 2.3. Lgr5 Expression Is Increased in Trp53 Tumors

Lgr5, ALDH1A, EPCAM, and CD44 mRNA levels were quantified by real-time PCR as part of a preliminary analysis of potential stem cell markers. No significant differences were detected between *Trp53 +/+* and *Trp53 −/−* tumors at the mRNA level (Figure S5). However, given that mRNA levels do not always directly correlate with protein expression, we decided to quantify Lgr5 protein levels, as it is a key stem cell marker in colon tissue and has been extensively validated for identifying colon stem cells, making it particularly relevant for our analysis.

**Figure 4:**
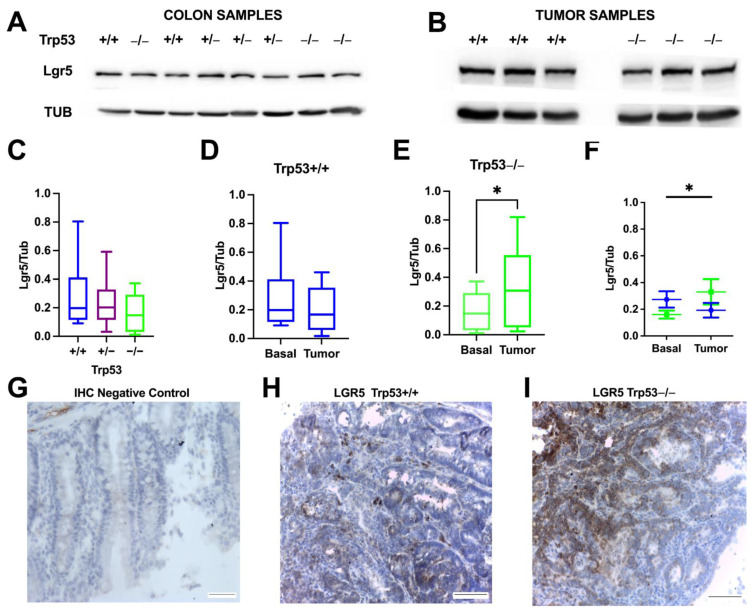
Difference in Lgr5 expression in p53 wild-type and knockout mice. (**A**) Representative Western blot membrane of the control animals. Lgr5 expression in controls with an indicated *Trp53* genotype. Tubulin was used as a loading control; (**B**) representative Western blot membrane of AOM/DSS-mice with an indicated *Trp53* genotype; (**C**) the percentile graphic shows the median of the basal expression (control animals) of Lgr5 in distal colons with indicated *Trp53* genotypes; (**D**) The percentile graphic compares Lgr5 in normal and tumor colons in *Trp53+/+*; (**E**) the percentile graphic compares Lgr5 in normal and *Trp53−/−* tumor tissue (T-Test * *p* < 0.05); (**F**) the graphic shows the mean and SEM comparing the basal and tumor expression of Lgr5 in *Trp53+/+* in blue (nine tumors) and *Trp53−/−* in green (nine tumors) (two-way ANOVA: interaction * *p* < 0.05); (**G**–**I**): comparison of Lgr5 staining by immunohistochemistry between tumor tissue of *Trp53+/+* and *Trp53−/−* mice; (**G**) negative control of the immunohistochemistry (reaction was carried out without the primary antibody); (**H**) Lgr5 staining of tumor tissue of *Trp53+/+* mice; (**I**) Lgr5 staining of tumor tissue of *Trp53−/−* mice. Scale bar = 100 µm. SEM (standard error of the mean).

Western blots were performed to quantify protein levels of stem cell marker Lgr5 in normal colon tissue in mice among genotypes (Figure 4A–F). In the control animals, Lgr5 basal levels were more variable in *Trp53+/+* mice compared to *Trp53−/−* mice (Figure 4C).

Lgr5 levels were relatively stable in both normal tissue and tumor samples from *Trp53+/+* mice, since there is no significant difference in LGR5 expression between normal and tumor tissue in *Trp53+/+* animals (Figure 4D). However, in *Trp53−/−* mice, there was an increase in Lgr5 expression in tumors compared to the non-tumor samples (Figure 4E). Comparing *Trp53+/+* and *Trp53−/−* genotypes, normal colons showed higher Lgr5 levels in *Trp53+/+* versus *Trp53−/−*. However, in the tumor samples, this was reversed, with higher Lgr5 levels observed in *Trp53−/−* compared to *Trp53+/+*. In summary, p53 knockout results in lower Lgr5 expression in normal colons but higher expression in tumor samples compared to *Trp53+/+* mice (Figure 4F).

To confirm that Lgr5 expression increased in tumor knockout mice, we performed immunohistochemistry for Lgr5 on colon tissue sections from wild-type and knockout p53 mice. As predicted by Western blot analysis, an increased Lgr5 staining was observed in tumor samples from knockout animals compared to wild-type animals (Figure 4H,I).

Given the increase in Lgr5, we investigated whether β-catenin expression was altered in our samples. Immunofluorescence was used to assess β-catenin levels between *Trp53+/+* and *Trp53−/−* animals. The staining patterns varied, showing membranous, cytoplasmic, and nuclear localization, with most adenocarcinomas exhibiting highly heterogeneous staining (Figure S6). By the time adenocarcinomas were abundant in both genotypes, there was no significant difference in β-catenin levels. Although *Trp53−/−* tumors showed a slight increase in β-catenin scores, this difference was not statistically significant (Figure S7). Both *Trp53+/+* and *Trp53−/−* tumors had similarly elevated levels of β-catenin staining in these heterogeneous tumors. 

### 2.4. Trp53 Tumors Showed Increased Levels of Laminin Tumor Tissue

Adenocarcinomas often produce excess mucin within tumor cells and glands and could be found in all three genotypes. Well-differentiated and moderately differentiated adenocarcinomas were frequent in *Trp53+/−* and *Trp53−/−*. Adenomas were abundant with different degrees of dysplasia and adenocarcinomas in the *Trp53+/+* (Figure S4). Since submucosal invasion sites were more easily found in *Trp53−/−* mice compared to other genotypes (Figure S5), we investigated vimentin expression as a marker of epithelial–mesenchymal transition (EMT) in tumors. Immunofluorescence on colorectal adenocarcinomas from *Trp53+/+* and *Trp53−/−* mice (Figure S8) showed a slight trend towards increased vimentin in *Trp53−/−* tumors, but no significant differences in epithelial vimentin staining were detected. These findings indicate a mesenchymal shift in tumors from both genotypes.

**Figure 5:**
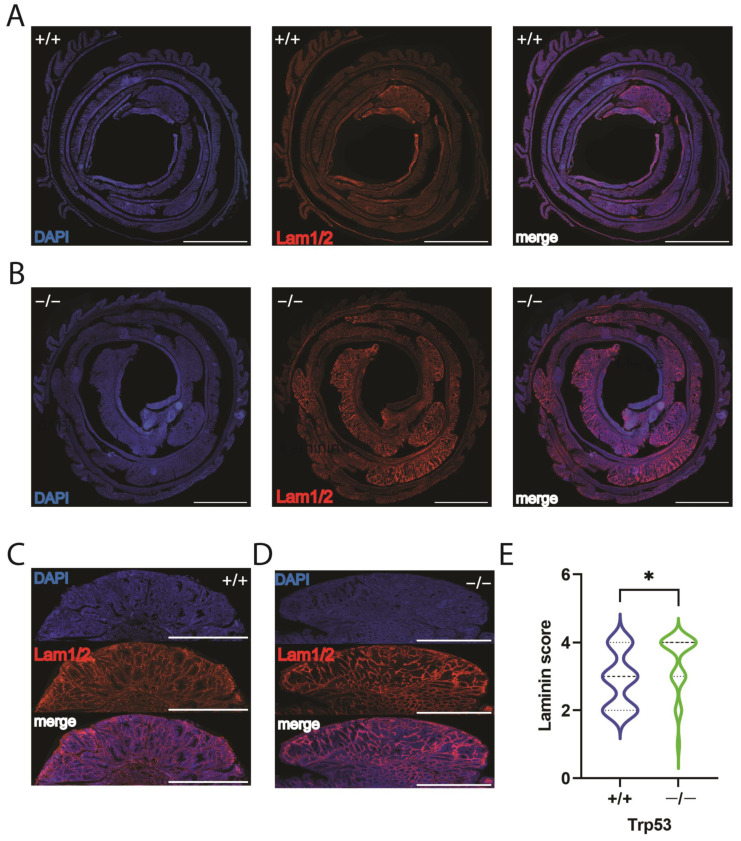
Trp53 tumors showed increased Laminin. (**A**,**B**) Wild-type and *Trp53* knockout colons were rolled as “Swiss rolls” and stained with nuclei staining 4′,6-diamidino-2-phenylindole (DAPI, blue) or laminin (red). (**A**,**C**) represents *Trp53+/+* and (**B**,**D**) represents *Trp53−/−* full “Swiss rolls” (bars 3 mm), and examples of a tumor and a peritumoral tissue (bars 1 mm). (**E**) Qualitative laminin intensity of tumoral areas according to the genotype; laminin immunofluorescence intensity was ranked as described in methods. *n* = 19 tumors from *Trp53+/+* and 28 *Trp53−/−*. * One-tailed Mann–Whitney *t*-test *p* < 0.05.

To analyze the effect of *Trp53* in colon tissue and tumors further, we analyzed laminin labeling by immunofluorescence. Laminin is a vital component of the basal membrane, and its barrier function is a critical first step leading to inflammatory diseases of the intestine [28]. In addition, lowering p53 levels in some stromal cells increased laminin expression, which increases tumor migration [11,12]. Therefore, we examined the levels of laminin in *Trp53+/+* and *Trp53−/−* in the whole colonic tissue, comparing tumor and non-tumor areas. As shown in Figure 5, *Trp53−/−* showed increased positive areas and augmented intensity staining as more tumors and larger tumor areas per colon were observed. Laminin immunofluorescence was increased in tumors compared to non-tumor mucosae (Figure 5A,B). *Trp53−/−* showed homogenous, more intense laminin staining compared to wild-type tumors (Figure 5C).

## 3. Discussion

This study reveals novel insights into how *Trp53* knockout influences colonic tumorigenesis and Lgr5 expression cells using the AOM/DSS mouse model of colitis-associated colon cancer. We demonstrate that *Trp53* loss exacerbates the colitis and tumor burden and reduces the latency of the Lgr5-positive adenocarcinomas. Deletion of *Trp53* led to increased expression of the stem cell marker Lgr5 in colorectal tumors compared to matched normal colon tissue. Supporting this conclusion, an elegant study demonstrated that specific deletion of *Trp53* in Lgr5-positive intestinal stem cells led to increased colorectal tumor size and incidence in the AOM/DSS model, though only a few mice were examined [22].

First, *Trp53* knockout mice showed more susceptibility to severe DSS-induced colitis, reflected in more prolonged diarrhea, bleeding, weight loss, and disease activity. This aligns with evidence that p53 helps resolve intestinal inflammation [29]. Persistent inflammation likely enabled more tumors in this group, supporting the notion that tumors from *Trp53−/−* could represent better IBD-related cancers than *Trp53+/+*. Agreeing with that idea, *Trp53−/−* tumor histology was prone to show invading adenocarcinomas that could be filled with mucin and increased laminin staining, characteristics related to IBD [30,31] and IBD-associated tumors [32]. *Trp53−/−* could also be a good model for DSS-only induced tumors [28], but most tumors induced by DSS alone were low-grade neoplasms, in contrast to the frequent submucosal invasion found here with the AOM/DSS protocol.

Our study adds critical discussion about “cancer latency”—the time between initial causal exposures and eventual cancer diagnosis. Some critics have emerged about this concept because cancer risk factors can be unpredictable and random [33]. This may be partly due to a lack of suitable model systems to study how risk factors contribute to the development of tumors over time, from the initial stages to progression to full-blown cancer. Our study looked at one significant risk factor for several cancers using a well-established model of colon cancer development. This allowed us to evaluate how this risk factor impacts tumor initiation and growth in a more controlled way than is possible in human studies. Our findings contribute to understanding cancer latency by showing how this risk factor drives tumor development in this colon cancer model.

Finally, *Trp53* deletion augmented Lgr5 marker expression in AOM/DSS induced- adenocarcinomas. Prior work indicates that p53 promotes stem cell differentiation [34]. Our finding of increased tumor Lgr5 levels with *Trp53* loss is also consistent with LGR5-positive cell expansion in human colorectal cancers [19,35]. Since Lgr5-positive cells can act as cancer stem cells (CSCs), their enrichment likely contributed to the knockout mice’s more aggressive tumor growth [17].

Studies on Lgr5 in CRC have produced disparate results, likely from the different models and systems used and Lgr5’s various functions. Lgr5’s role likely varies considerably depending on context. Despite controversies, Lgr5 has an essential role at specific points during CRC development or progression. Notably, there appears to be consensus on Lgr5’s role in CRC cell survival and metastatic progression [36].

Moreover, in the *Trp53−/−* group, the increased tumor level of Lgr5 expression agrees with most observational studies, suggesting a positive role for LGR5 expression in CRC progression [37,38]. Our *Trp53−/−* results indicate expanded Lgr5-positive cells during tumorigenesis. With only p53 reduction, increased CSCs can occur, suggesting that p53 loss enables a stem-like state [39]. However, other studies found Lgr5 downregulation during CRC progression, returning in metastases [36].

Our results highlight the complex interaction between p53 loss, Lgr5 expression, and tumor progression markers in the AOM/DSS-induced colorectal carcinogenesis model. Despite a significant selective increase in Lgr5 expression in *Trp53−/−* tumors, we did not observe corresponding changes in β-catenin levels or distribution between the *Trp53+/+* and *Trp53−/−* genotypes. Although a slight increase in β-catenin scores was noted in *Trp53−/−* malignant tumors by immunofluorescence, both *Trp53+/+* and *Trp53−/−* exhibited increased β-catenin expression and cytoplasmic-to-nuclear translocation in highly heterogeneous tumor populations. The lack of β-catenin differences between adenocarcinomas from *Trp53+/+* and *Trp53−/−* mice might be attributed to the advanced stage of the tumors analyzed. Previous studies, such as those by Clapper ML [40], reported β-catenin alterations primarily in polypoid dysplasias, an earlier stage of tumorigenesis. By focusing on adenocarcinomas in this study, we may have missed the window where β-catenin signaling differences are more pronounced. While p53 loss accelerates tumor development, our findings suggest that by the time tumors reach the adenocarcinoma stage, β-catenin levels may converge, regardless of p53 status.

The increased Lgr5 expression in p53 knockout tumors may also come from non-Lgr5+ crypt-base sources. While mutated intestinal Lgr5-positive stem cells are an established origin of cancer [15,16], other sources exist. More differentiated transit-amplifying progenitors may reacquire Lgr5 expression and become cancerous [41]. Additionally, oncogenic mutations could drive Lgr5-positive cancer cells from enterocyte or secretory precursor dedifferentiation toward a stem-like state [41]. There is also evidence of Lgr5-positive cell generation through Lgr5-negative tumor cell plastic transitions [42].

Our findings highlight the intricate relationship between *Trp53* deletion and colorectal tumor dynamics, marked by an increased tumor burden and shorter latency periods in Lgr5-positive adenocarcinomas. This acceleration in tumor development may be partly driven by enhanced inflammation resulting from the loss of p53’s regulatory effects on cellular processes. In the absence of the p53 wild-type function, there is an increased expression of IL-6 and NF-κB activity [41,43,44,45]. However, the increase may differ if inflammation is chronic, acute, associated with a carcinogen, or even dependent on which type of cell is deficient in p53. Spehlmann et al. (2013) [44] contributed significantly to our understanding by demonstrating that p53-deficient macrophages and dendritic cells exhibit increased IL-6 and NF-kB activity expression in response to various inflammatory stimuli [44]. Similarly, Marruecos et al. (2021) revealed that losing a single *Trp53* allele in macrophages leads to increased inflammatory responses mediated by regulating IκBα. These findings underscore the importance of p53 in modulating inflammatory responses in immune cells [43].

Schwitalla et al. (2013) [41] showed that whereas p53-deficient intestinal epithelial cells increase in survival during the initiation stage, the loss of p53 during tumor progression is associated with increased intestinal permeability, causing the formation of an NF-kB-dependent inflammatory microenvironment [41]. This initial protection is consistent with Spehlmann et al. (2013) [44], which was observed after acute inflammation and the initial damage in the colon tissue induced by AOM treatment [44].

In our model, which involves repeated low doses of DSS combined with a single dose of AOM, the deletion of p53 initially provided a protective effect against AOM, as evidenced by a reduced disease activity index (DAI) in the early days (Figure 2D). However, this protection was abruptly followed by a rapid onset of severe colitis after DSS exposure, characterized by sudden weight loss, bleeding, diarrhea, and increased DAI. By the time malignant tumors were abundant across all genotypes, no significant differences in inflammation markers were detected via qPCR, suggesting that the inflammation in *Trp53−/−* mice was likely temporary. Although increased immune cell infiltration was still observed in tumors, the lack of significant intestinal inflammation indicates a transient inflammatory response. A detailed temporal study could further clarify these dynamics, but such an investigation would require a large number of animals and is beyond the scope of this proposal. These findings point to a complex interaction between p53, inflammation, and tumorigenesis. Despite the initial protective effect, the absence of wild-type p53 eventually led to an increased tumor burden, highlighting p53’s dual role in regulating both epithelial apoptosis and inflammation in colorectal cancer progression [28,41,45,46].

## 4. Materials and Methods

### 4.1. Mice and Genotyping

Transformation-related protein 53 (*Trp53*) *+/−* mice were initially obtained from the Jackson Laboratory. The mice were backcrossed for ten generations into the 129S6SvEvTac background and once with C57BL/6 to reduce the testicular teratomas associated with the *Trp53−/−* mice in the 129 background [47]. They were housed and bred in the Mouse Facility of the Institute of Biomedical Sciences of the Federal University of Rio de Janeiro. The animals were kept in ventilated microisolator cohousing cages (Alesco, BR) with free access to filtered tap water, premium pellet food, and environmental enrichment. Mouse rooms were maintained at approximately 25 °C with a 12 h light-dark cycle. *Trp53+/−* and *Trp53−/−* male mice were mated with *Trp53+/−* and *Trp53+/+* females to generate offspring of all genotype variants. An ear punch numerical system identified all offspring between days 21–30 after birth. Genotyping was performed as in [48].

### 4.2. AOM/DSS Colorectal Cancer Model

*Trp53+/+* (*n* = 22), *Trp53+/−* (*n* = 20), and *Trp53−/−* mice (*n* = 28), including males and females, with a median age of 10 weeks old, were pretreated with Vetmax Plus liquid (as indicated by the veterinarian) three days before the AOM/DSS experiment (Figure 1, upper panel). To avoid bias during measurement and equalize potential environmental influences, animals of the same sex but different genotypes were assigned unique numeric identifiers and co-housed whenever feasible. All mice were weighed and received an intraperitoneal injection of azoxymethane (AOM; Sigma Aldrich, St. Louis, MO, USA, EUA A5486) diluted in sterile saline (12.5 mg/kg). Five days after the AOM injection, the colonic inflammatory agent sodium dextran sulfate (DSS, 0216011080; MP BIO, Santa Ana, CA, USA; molecular weight 36,000–50,000 Da) was diluted (2%) and added to filtered drinking tap water that was provided ad libitum for five days. The second DSS cycle was also for five days, but 1.5% of DSS was used. In the last cycle, 1.5% DSS was given for four days (ethics committee at the Care and Use of Animals of the Federal University of Rio de Janeiro, 0085/15). The health and welfare of all mice were assessed daily during the AOM-DSS protocol. All animals were weighed and examined for diarrhea, rectal bleeding, rectal prolapse, and body curvature. Behaviors indicating pain or distress resulted in immediate euthanasia as a humane endpoint criterion. Pre-established criteria for early endpoint euthanasia of animal subjects included weight loss greater than 10% in two consecutive days, reduced spontaneous ambulatory activity, abnormal posture, lack of grooming, and other observable indicators of pain or distress. Regardless of weight loss parameters, an animal displaying significant changes in behavior or appearance indicative of morbidity, pain, or discomfort was humanely euthanized before the end of the planned study duration per protocol guidelines. *Trp53−/−* animals had to be euthanized two weeks earlier (day 67) than *Trp53+/−* and *Trp53+/+* animals (day 89), as they had already developed multiple colorectal tumors (eventuated by colonoscopy) and showed signs of discomfort. Unless otherwise stated, the final number of animals of the experiment in the figures was *Trp53+/+* (13), *Trp53+/−* (14), and *Trp53−/−* (8). The final efficiency rate of the experiment, measured by the number of macroscopic tumors in the intestines after euthanasia, *Trp53−/−* mice had a 100% tumor efficacy, even with the colons removed 20 days earlier than the *Trp53+/+* and *+/−* groups.

### 4.3. Disease Activity Index

The Disease Activity Index (DAI) was determined daily for each animal based on clinical observations. The parameters of percentage weight loss, diarrhea, and bright red blood through the rectum or in the stool (hematochezia) were evaluated and scored according to the criteria outlined in Table S1. The DAI score for each animal was calculated as the sum of the scores for these three parameters. The mean DAI was determined for each experimental group by calculating the total DAI for all animals divided by the number of animals in that group. This calculation provided a daily average DAI score for each treatment group. This index was modifiable by the previously described method [49].

### 4.4. Endoscopic Evaluation of Colonic Inflammation and Tumorigenesis

The colonoscopy was performed as in [50]. Briefly, a pediatric flexible bronchofiberscope (FB120P; Fujinon, Tokyo, Japan) was used on mice anesthetized with isoflurane (Cristália; São Paulo, Brazil) at 1.5% in 1.5 L/min oxygen, using a laboratory animal anesthesia system (EZ-7000; Euthanex, Palmer, PA, USA). The mice were kept supine over a stainless steel heated surgical waterbed at 37 °C using the T/Pump System (Gaymar, Orchard Park, NY, USA). An enema was performed with 1 mL of water to remove feces before the introduction of the endoscope. Colonoscopy images were captured and stored, and aspects of the colons, such as wall transparency and bleeding, were taken during the procedure. The presence of focal lesions, or tumors, was registered for each animal. Based on these findings, a distal–proximal endoscopic colitis scoring system was adopted (Figure S1) modified from [51].

### 4.5. Labeling Macroscopic Big Tumors

AOM/DSS-treated mice were euthanized according to the Ethics Committee on Animal Care and Use Guidelines of the Federal University of Rio de Janeiro. The rectums and colons were opened longitudinally, and stool was removed. The opened large intestines were photographed with a Canon 60D digital camera or Samsung S10 cell phone. The larger tumors (2–4 mm) were half-cut with surgical blades and identified with pigment liquid ink (Xadrez, Sherwin Williams, Cleveland, OH, USA). Up to 4 tumors per colon were labeled for matching macroscopic biopsies with histological samples. Tumor biopsies (1–2 mm fragments) were snap-frozen in liquid nitrogen and stored −80° C for qPCR and Western blot analysis. The colons were wrapped in a disposable plastic micro pestle to form a “Swiss roll.” The tissues were fixed in 4% paraformaldehyde for 24 h and subsequently processed for paraplast embedding.

### 4.6. Histological Processing of Samples from the Colons of Animals

“Swiss roll” colons were fixed in 4% paraformaldehyde for 24 h and then transferred to 70% ethanol. The tissues passed through one incubation of 80%, 90%, and 100% ethanol for 15 min each at room temperature and one with 100% isopropyl alcohol for 15 min at room temperature. After isopropyl alcohol incubation for 15 min at 65 °C, the tissues were transferred to isopropyl alcohol and paraplast (Sigma-Aldrich, St. Louis, MO, USA); 145686-99-3) melted in a 1:1 ratio for 30 min at 65 °C, followed by two incubations in 100% paraplast for 1 h each at 65 °C. The tissues in paraplast blocks were sectioned in a microtome (Leica RM2235) into 5 µm slices and positioned on slides previously treated with poly-L-lysine (350 µg/mL—Sigma-Aldrich; P2636).

### 4.7. HE Staining

The slides were deparaffinized by heating in a drying oven at 60 °C for 20 min and then immersed in three 5 min xylene baths. The slides were then hydrated with 5 min immersions in 100%, 90%, 80%, and 70% alcohol and distilled water. For staining, Harris Hematoxylin (Easypath, São Paulo, Brazil) was applied for 2 min; then, the slides were placed in running water for 5 min. The slides were dipped twice in hydrochloric alcohol (1:50 dilution), followed by two Eosin (Easypath) dips with 1% acetic acid, and finally distilled water. For dehydration, the slides underwent the reverse sequence of hydration and deparaffinization: distilled water, 70% alcohol, 80% alcohol, 90% alcohol, 100% alcohol, and three 1 min xylene immersions. Finally, the slides were mounted with D.P.X (Sigma STBC5140v).

### 4.8. Histological Tumor Analysis

High-grade dysplastic colorectal tumors were evaluated by architectural complexity, cribriform glands, mucin accumulation, and nuclear features consistent with high-grade adenoma or adenocarcinoma. Priority was given to adenocarcinomas with evident infiltrating growth and irregular glandular structures. Well-differentiated and moderately differentiated adenocarcinomas from all genotypes were included, with or without excess mucin production. Adenocarcinoma diagnosis was confirmed by histopathology (Chagas, V) across all three genotypes. All frozen tumor biopsies used for blotting analysis underwent prior histological examination to ensure at least moderate dysplasia.

### 4.9. Immunohistochemistry

Five µm thick sections were heated at 60 °C for 20 min, followed by five minutes of incubation in xylene 100% three times. The samples were rehydrated by immersion in decreasing concentrations of ethanol (100%, 90%, 80%, and 70%), distilled water, and finally, PBS for 5 min each. Heat-induced antigen retrieval lasted 40 min at 95 °C using EnVision FLEX Target Retrieval Solution, High pH (DM828) from the EnVision Flex High Ph Link Kit (Dako, K8000, Carpinteria, CA, USA). The slides were cooled for 20 min in tris-buffered saline with 0.1% Tween at room temperature. The slides were then incubated with 0.5% Triton and three washes with TBS-T for five minutes. The EnVision FLEX peroxidase blocking reagent (SM801) was used for 25 min in a darkroom to block peroxidase. Non-specific antigen binding was blocked by a one h incubation with 5% bovine serum albumin (Bovine Serum Albumin—BSA) diluted in TBS. The primary antibodies Anti-LGR5 (Abcam-ab75850, 1:200 in TBS 1% BSA, Cambridge, England) were incubated at room temperature and protected from light for one h. Primary incubation was followed by three 5 min washes with TBS-T 0.1% and incubation with the ready-to-use secondary from the Dako EnVision FLEX/HRP kit (SM802) for 45 min in a dark chamber and washed with TBS-T. Staining was developed with 3,3;’diaminobenzidine (DAB) for 60 s in a drop of EnVision FLEX DAB Chromogen (DM827) diluted in 1 mL of EnVision FLEX Substrate Buffer (SM803), washing with distilled water to stop the reaction. Counterstaining was performed with filtered Hematoxylin (EasyPath) for two minutes. All slides were sealed with Gelvatol (Polyvinyl Alcohol Sigma-341584, Glycerol, 0.2 M Tris HCl pH 8.5, H_2_O) and with nail polish.

### 4.10. Immunofluorescence

Five µm thick sections were heated at 60 °C for 20 min, followed by five minutes of incubation in xylene 100% three times. The samples were rehydrated by immersion in decreasing concentrations of ethanol (100%, 90%, 80%, and 70%), distilled water, and finally PBS for 5 min each. Heat-induced antigen retrieval lasted 20 min at 95 °C using 10 nM of Sodium Citrate Buffer 0.05% Tween 20 at a pH of 6,1. Three washes with PBS for five minutes. Ammonium chloride 50 mM 0.5% Triton diluted in PBS was used for 10 min. Wash three times with PBS for five minutes. Normal goat serum (NGS) 5% was blocked by one-hour incubation and diluted in PBS for laminin and Bovine Serum Albumin (BSA) 5% was blocked by one-hour incubation and diluted in PBS for β-catenin and vimentin. Primary antibodies anti-laminin (Abcam-ab7463, 1:200 in PBS NGS 1%), anti-vimentin (Cell Signaling-5741, 1:50 in PBS BSA1%), and anti-β-catenin (Abcam-ab16051, 1:100 in PBS BSA 1%) were incubated overnight at a 4 °C temperature and protected from light. Followed by three 5 min washes with PBS and incubation in goat-anti-Rabbit 1:500 (Abcam-A11010) for laminin and goat-anti-Rabbit 1:400 (Invitrogem-A11008) for 2 h in a dark chamber. After three washes with PBS for five minutes each, the slides were stained with nuclei staining 4′,6-diamidino-2-phenylindole (DAPI). All slides were sealed with ProLong (Invitrogen-P36930, Waltham, MA, USA) and covered with coverslips.

### 4.11. Immunofluorescence Analysis

Tissue slides were evaluated independently by at least two authors. The intensity scoring of Laminin is based on the immunoreaction score (IRS) [29] and was performed separately by the coauthors AFC and JMD to compare tumors from *Trp53 +/+* and *Trp53 −/−*. Morphological features and labeling intensity were assessed. Semi-quantitative scoring categorized staining as “strong (4)”, “moderate (3)”, “weak (2)”, or “absent (1)” in epithelial or tumor tissues [30].

The analysis of β-catenin expression in tumors was performed at both the cell level and the overall tumor level. For cells, a four-level system was used: (1) weak staining limited to the membrane with clear cell boundaries; (2) strong membrane staining; (3) stronger membrane staining with some cytoplasmic involvement; and (4) intense staining across the membrane, cytoplasm, and nucleus, making cell boundaries hard to see. For the overall tumor, heterogeneity was considered: in mixed tumors, the two main areas were analyzed separately, and their scores were added; in uniform tumors, the score was doubled. If there were three or more different areas, only the two highest were counted. The final score ranged from 2 to 8.

Vimentin expression in tumors is assessed using a five-point scale from 0 to 4, focusing only on epithelial regions, with any staining in the submucosa and lamina propria being ignored. A score of 0 means no vimentin staining in the epithelial cells, indicating no expression. A score of 1 indicates weak staining, with about 10% of the epithelium showing vimentin expression. Score 2 is given for moderate staining in the epithelium. Score 3 represents strong staining in about 50% of the epithelial cells. The highest score, 4, is assigned when intense staining is seen in more than 50% of the epithelium, indicating widespread vimentin expression.

### 4.12. Tissue Protein Extraction

Distal colons from control mice *Trp53+/+* (*n* = 13), *Trp53+/−* (*n* = 14), and *Trp53−/−* (*n* = 8) and distal tumor samples were weighed using a precision scale. Dried frozen fragments were macerated in microtubes with disposable plastic micro pestles and liquid nitrogen. An amount of 60 mL of lysis buffer (7 M Urea, 2 M Thiourea, 1% DTT, 0.8% NP40, 1% DOC, 75 mM Tris-HCl pH 7.5) per gram of tissue was added and kept for 15 min. Each sample was sonicated three times on ice for five pulses and then centrifuged at 14,000× *g* for 15 min at 4 °C. Supernatants were collected and frozen at −80 °C.

### 4.13. Western Blotting

The samples were applied on a 10% acrylamide gel, initially run at 40 V for 50 min and then 120 V for 1 h and 50 min. The transfer occurred overnight in the Towbin buffer (25 mM Tris, 192 nM Glycine, pH 8.3) for 15 h and 30 min at 40 V. PVDF membranes were cut, and fragments were placed in a 15 mL conical tube with a blocking solution with 5% BSA in TBS-T 0.1% at room temperature for 1 h on a rotary shaker. The primary antibodies were diluted in 5 mL of TBS-T 0.1% as follows: mouse anti-tubulin (Sigma Aldrich-67, 1:20,000), rabbit anti-Lgr5 (Abcam-ab75850, 1:5000) incubated overnight at 4 °C. Excess antibody was removed with a serological pipette; membranes were washed three times in 0.1% TBS-T and incubated for one hour at room temperature with 1:20,000 anti-mouse and anti-rabbit secondary horseradish peroxidase antibodies (1:10,000) in TBS-T 0.1%. Image acquisition was performed in ChemiDoc (Biorad, Hércules, CA, USA) with Immobilon substrate (Biorad).

### 4.14. Statistical Analysis and Manuscript Editing Assistance

Statistical analysis and English grammar assistance were performed using GraphPad Prism 9.0.0 (San Diego, CA, USA) and OpenAI’s ChatGPT (San Francisco, CA, USA, EUA), respectively.

### 4.15. Length of Colon

The colon lengths were consistently measured using images taken during euthanasia and analyzed with ImageJ software v1.53t. The graph displays the length of the colon from the rectum to the start of the proximal colon, where typical folds are visible.

### 4.16. Histological Inflammation Scores

The Histological Inflammation Score (HIS) for the “Swiss roll” colon sections was assessed based on three parameters: (1) epithelial changes (prolonged epithelial cell or crypt alterations = 1 point; barrier disruption = 2 points; ulceration of < 50% of the area = 3 points; ulceration of > 50% = 4 points); (2) immune cell infiltration (mild = 1 point; moderate = 2 points; severe = 3 points); and (3) submucosal immune cell infiltration (mild = 1 point; moderate = 2 points; severe = 3 points). The total HIS was the sum of these parameters [52,53].

The Histological Inflammation Score for adenocarcinomas (HISa) was determined via a blinded analysis of the colons from the animal subjects. Inflammation severity was independently graded as minimal (1), mild (2), moderate (3), severe (4) for both tumor mass and adjacent submucosa regions. The HISa was computed as the aggregate of inflammation scores for the tumor and its adjoining submucosa. In cases of multiple tumors within the colon, the HISa was derived as the average of each tumor and its corresponding adjacent submucosa analysis.

### 4.17. RNA Extraction and cDNA Synthesis

RNA from 19 samples (*Trp53+/+ n* = 10 and *Trp53−/− n* = 9), stored in the freezer at −80 °C since 2017, weighing between 1 and 14.2 mg, was extracted using 0.5 µL of Trizol Reagent (Invitrogen, 15596-026, Waltham, MA, USA), according to the manufacturer’s instructions. The extracted samples were quantified using the Nanodrop 2000 (Thermo Fisher Scientific, Waltham, MA, USA) and stored at −80 °C until use. cDNA synthesis was performed using the High Capacity cDNA Reverse Transcription kit (Applied Biosystems, Waltham, MA, USA) in a GeneAmp PCR System 9700 (Applied Biosystems) thermal cycler, with nuclease-free water, resulting in a final volume of 50 µL of cDNA.

### 4.18. Real-Time PCR

For real-time PCR, in each well, 1 µL of cDNA and 19 µL of a mix containing Power SYBR Green PCR Master Mix (Applied Biosystems, 4367659), nuclease-free water, and specific primers (Table S2) or the reference primer (GAPDH) were plated in a 96-well PCR microplate (Axygen, Union City, CA, USA, 321-63-051). For each primer of interest, the samples were plated in duplicate and normalized with GAPDH. Amplification was performed under the following conditions: 50 °C for 2 min, 95 °C for 10 min, followed by 45 cycles at 95 °C for 15 s and 60 °C for 1 min. Real-time PCR results were acquired and analyzed using the 7500 Real-Time PCR System software v2.3 (Applied Biosystems).

## 5. Conclusions

The results showed the exacerbating effect of *Trp53* deletion on colitis and its facilitation of cancer stem cell expansion, accelerating tumorigenesis in a mouse model of CRC. This study significantly contributes to the understanding of the molecular dynamics of colorectal cancer, particularly in the context of inflammation-driven mechanisms. This IBD-associated cancer model might aid inflammation-driven tumorigenesis and therapeutic development analyses.

## Data Availability

The original contributions presented in the study are included in the article/Supplementary Material; further inquiries can be directed to the corresponding author.

## Supplementary Materials

The following supporting information can be downloaded at: https://www.mdpi.com/article/10.3390/ijms252010953/s1.
