# Supplementary material for: Trp53 Deletion Promotes Exacerbated Colitis, Facilitates Lgr5+ Cancer Stem Cell Expansion, and Fuels Tumorigenesis in AOM/DSS-Induced Colorectal Cancer
Source: Int J Mol Sci. 2024 Oct 11;25(20):10953. doi: 10.3390/ijms252010953 (PMC11507199; doi:10.3390/ijms252010953)
Supplement: Supplementary file 1 [file ijms-25-10953-s001.zip › ijms-3244888-supplementary.pdf]

Supplementary material, Cunha et al 2024

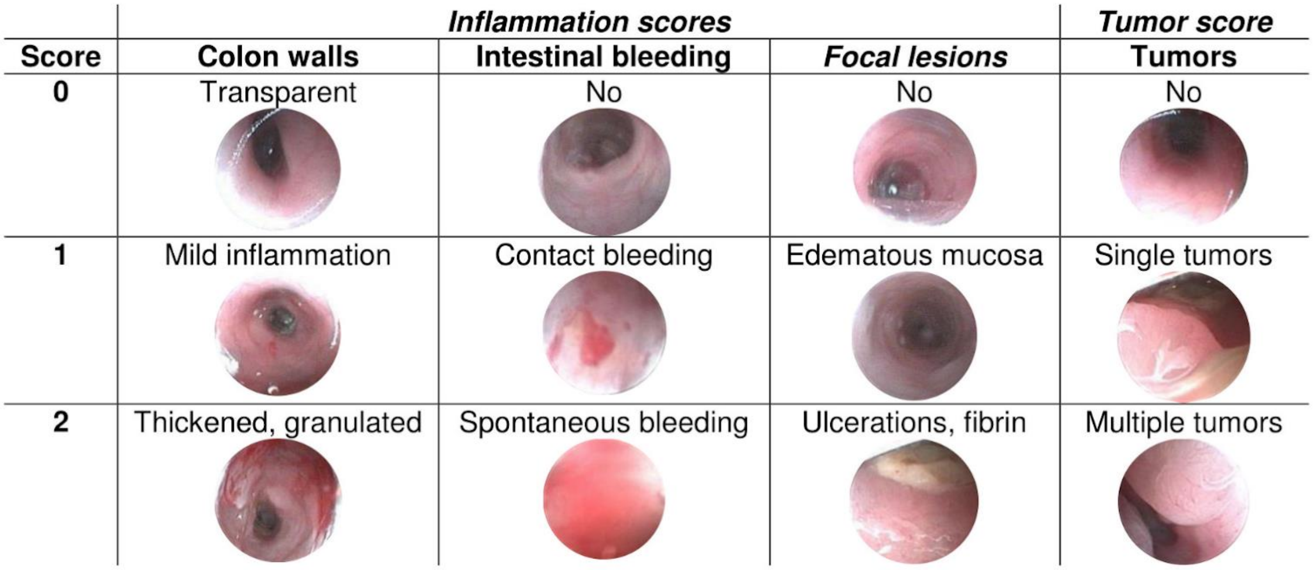

S1 Figure: *Endoscopic evaluation of colonic inflammation and tumorigenesis evaluation with representative images.*

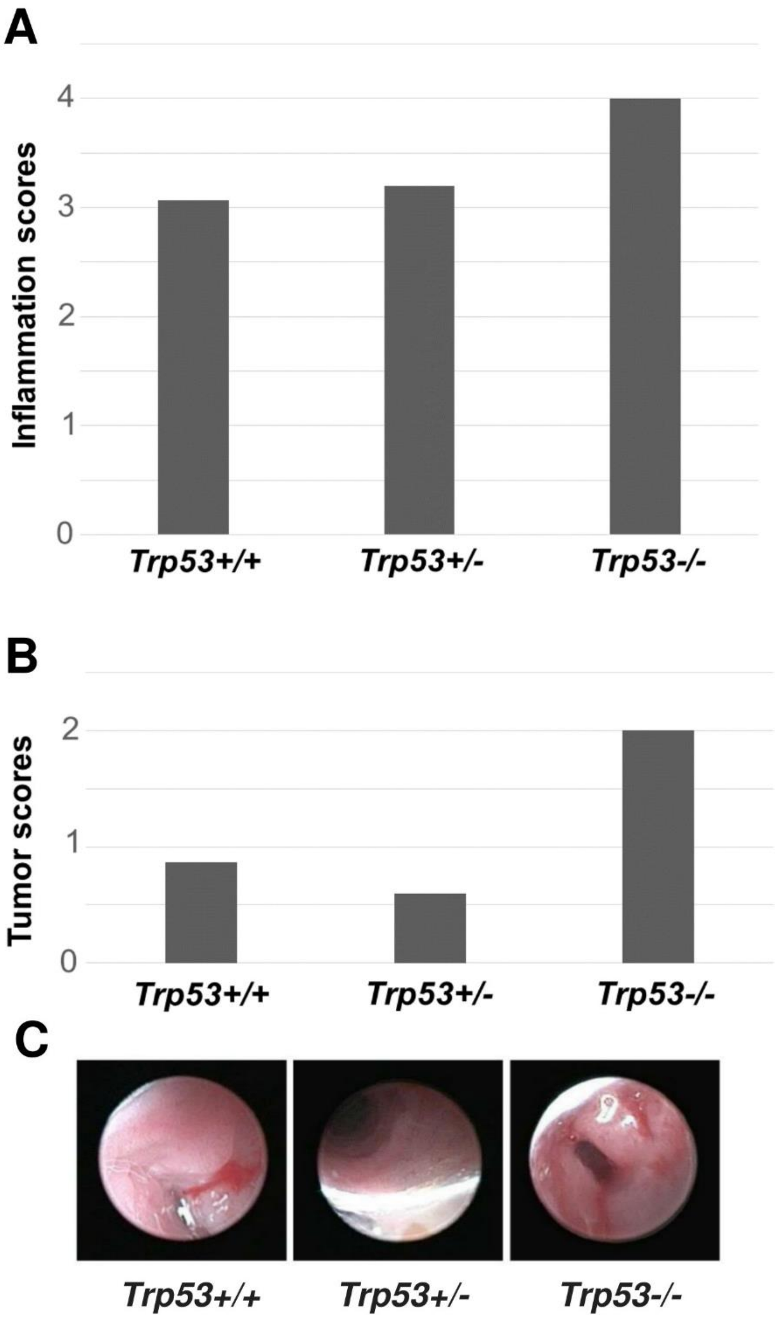

S2 Figure. *Colonic endoscopic evaluation of inflammation and tumorigenesis in the AOM/DSS model by Trp53 genotype. A) Inflammation scores, B) tumor scores, and C) representative colonoscopy images at nine weeks. Data shown as mean for Trp53+/+ (n=15), Trp53+/- (n=10), and Trp53-/- (n=2) genotype groups.*

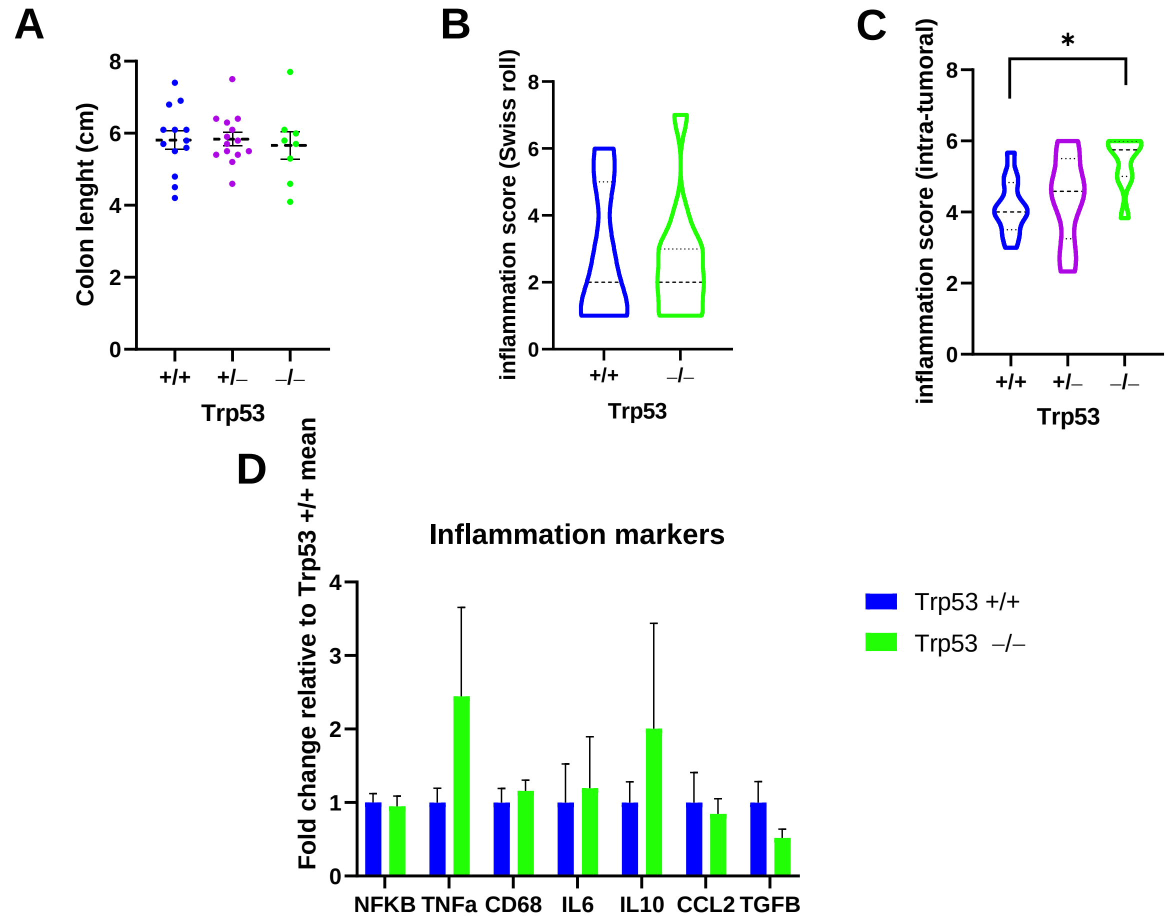

S3 Figure. _Colon length and inflammation score across the indicated genotypes. a) The graphic shows the m.s.e of the rectum, distal, and mid-colon length according to the genotype after completing the AOM/DSS model protocol. (One-Way ANOVA: *$p<0.05$). b) Violin plot showing the distribution of the inflammation score of the colon "swiss-roll" performed as described in the methods with the indicated genotypes c) Violin plot showing the distribution of the inflammation score intra-tumoral among the indicated genotypes. The relative amount of immunological cell infiltration in tumors was evaluated as described in methods (One-Way ANOVA: *$p<0.05$). d) Relative expression of inflammation markers in Trp53+/+ (n=10) and Trp53-/- (n=9) mice performed by qPCR. Expression levels of some inflammation markers (NFKB, TNFa, CD68, IL6, IL10, CCL2, TGFB) relative to Trp53+/+ control. Data are presented as the mean ± standard error of the mean. Two-way ANOVA analysis revealed no statistically significant differences between groups ($p>0.05$)._

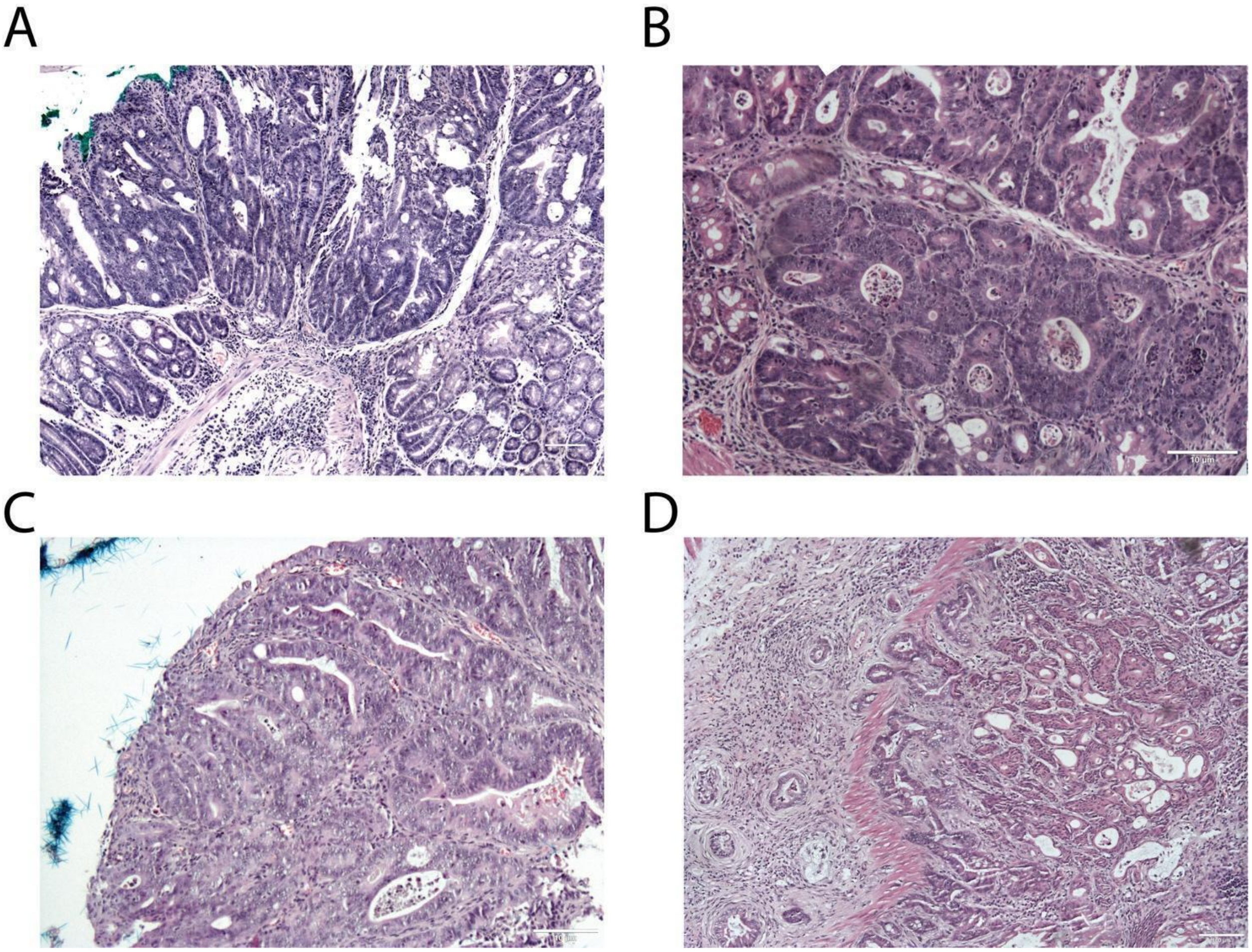

S4 Figure. *Histology of typical tumors found according to the genotypes. Photomicrographs of 5 μm sections of paraplast-embedded tissue stained with hematoxylin and eosin from colorectal samples of AOM/DSS. A) tubular adenoma Trp53+/+, B) adenocarcionma Trp53+/+, C) adenocacinoma Trp53+/-, D) typical submucosal invasion of adenocarcinoma Trp53 -/-. Scale bar A), B), C) = 10um, D) = 100um.*

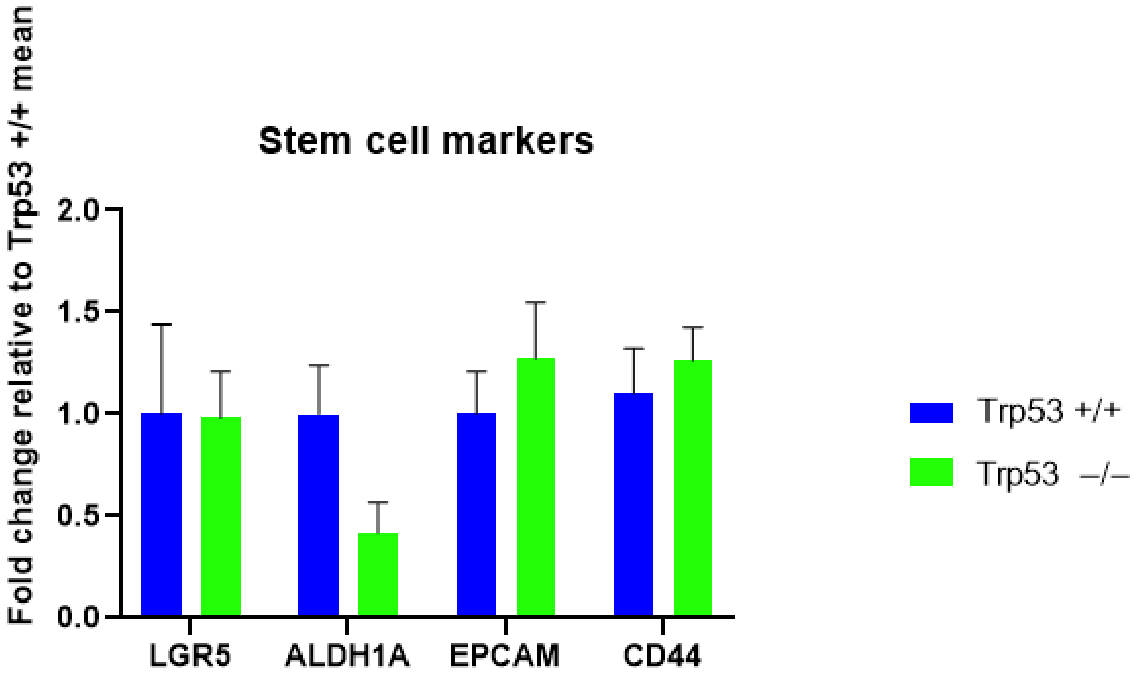

S5 Figure. Relative expression of stem cell markers in Trp53+/+ (n=10) and Trp53-/- (n=9) mice performed by qPCR. Expression levels of stem cell markers (LGR5, ALDH1A, EPCAM, CD44) relative to Trp53+/+ control. Data are presented as the mean ± standard error of the mean. Two-way ANOVA analysis revealed no statistically significant differences between groups (p>0.05).

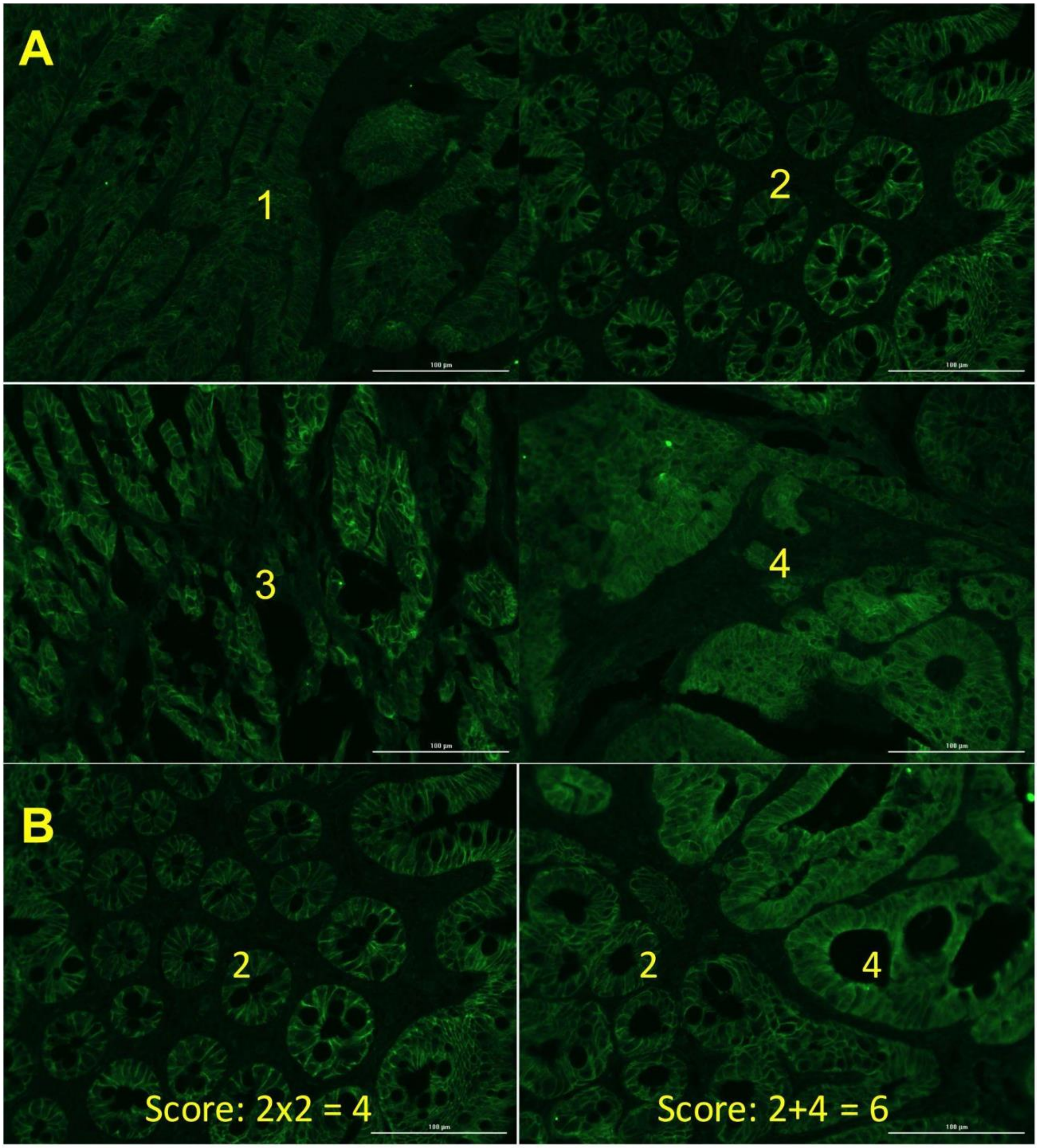

S6 Figure β-catenin score. A) Examples of tumor areas classified according to the scoring system described in the methods. Numbers (1-4) indicate different levels of β-catenin expression and localization. (B) Demonstration of total score calculation for homogeneous (left) and heterogeneous (right) tumors, as explained in the methods.

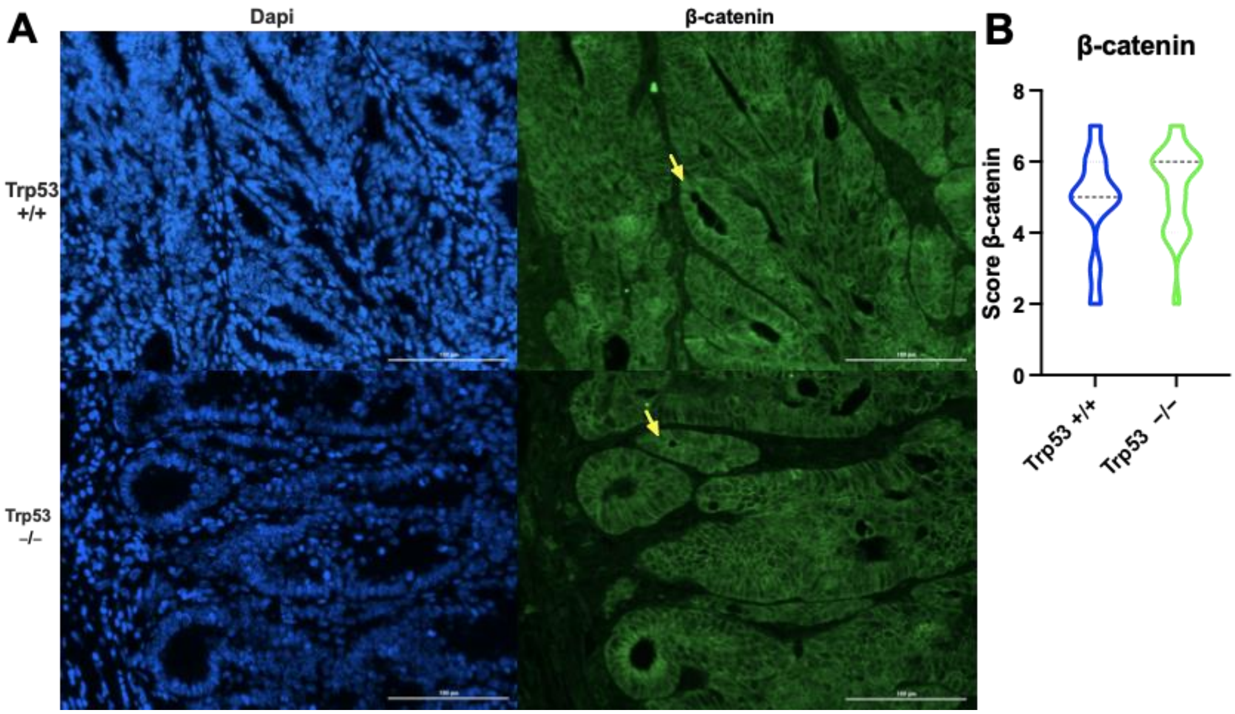

S7 Figure. Analysis of β-catenin expression in Trp53+/+ and Trp53-/- mouse tumors. (A) Representative images of 4',6-diamidino-2-phenylindole (*DAPI blue) and β-catenin (green) staining in Trp53+/+ (upper panel) and Trp53-/- (lower panel) tumors. Yellow arrows point to regions considered score 4 Scale bar: 100 µm. (D) Violin plot showing the distribution of β-catenin scores in Trp53+/+ (n=10) and Trp53-/- (n=9) tumors. Statistical analysis was performed using the Mann-Whitney test, resulting in (p>0.05).*

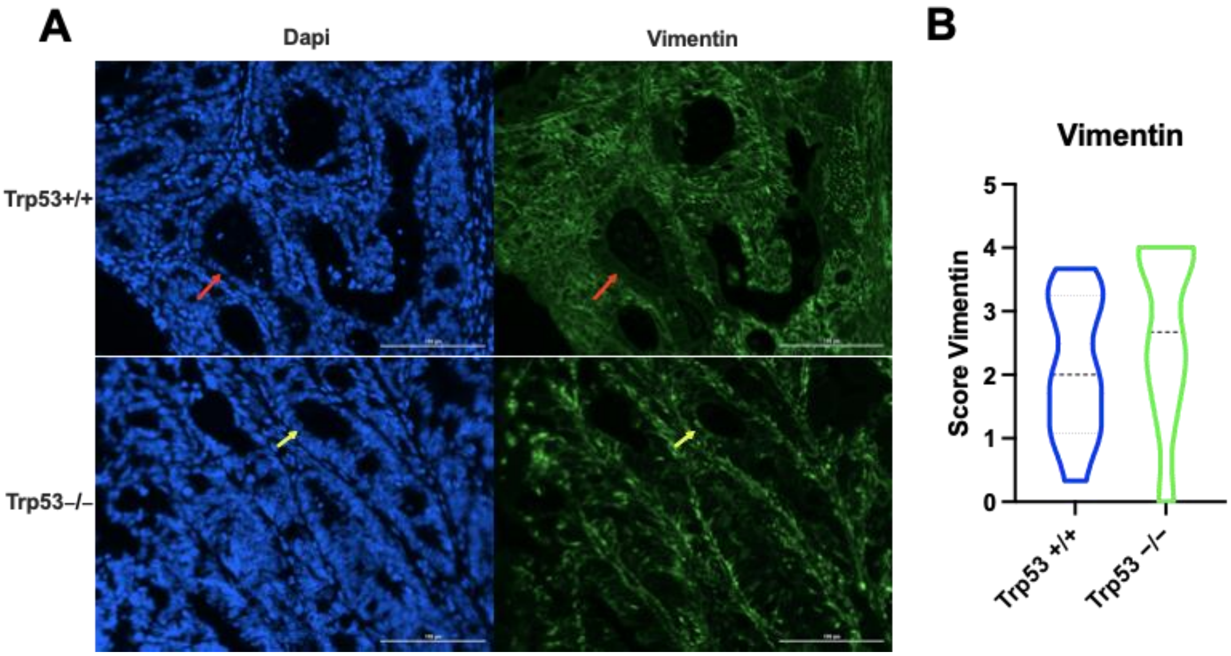

S8 Figure. *Vimentin expression in Trp53+/+ and Trp53-/- mouse colorectal adenocarcinomas***.** *(A) Representative immunofluorescence images of colorectal adenocarcinomas from Trp53+/+ (upper panels) and Trp53-/- (lower panels) mice. Red arrows indicate vimentin-negative epithelium, while yellow arrows highlight vimentin-positive epithelium. Nuclei are stained with* 4',6-diamidino-2-phenylindole (DAPI, blue), *and Vimentin expression is shown in green. Scale bars: 100 µm. (B) Quantification of Vimentin expression was specifically performed in the epithelial tissue of Trp53+/+ (n=12) and Trp53-/- (n=9) tumors, excluding the lamina propria and submucosa where vimentin is characteristically expressed. The scoring was evaluated as described in the Methods section. Statistical analysis using an unpaired t-test showed no significant difference in vimentin expression between the two groups ($p > 0.1593$).*

SCORE	Weight Loss	Diarrhea	Hematochezia
0	0%	Absence	Absence
1	]0%, 10%]		
2	]10%, 15%]	Presence	
3	]15%, 20%]		
4	]20%, +∞ [		Presence
DAI = Weight Loss + Diarrhea + Hematochezia			

Table S1. The Disease Activity Index (DAI) was determined daily for each animal based on clinical observations.

Primer	Sequence F	Sequence R
**ALDH1A**	TGTTAGCTGATGCCGACTTG	TTCTTAGCCCGCTCAACACT
**Ccl2 (MCP-1)**	CAGCCAGATGCAGTTAACGC	GCTTCTTTGGGACACCTGCT
**CD44**	TAGGAGAAGGTGTGGGCAGAA	GAGCTCACTGGGTTTCCTGTCTT
**CD68**	GCTGTGGAAATGCAAGCATAG	GAGAAACATGGCCCGAAGT
**EPCAM**	TTGCTCCAAACTGGCGTCTA	ACGTGATCTCCGTGTCCTTGT
**GAPDH**	CAACTTTGTCAAGCTCATTTCCT	CCTGTTGCTGTAGCCGTATT
**IL-10**	TGCTAACCGACTCCTTAATGCAGGAC	CCTTGATTTCTGGGCCATGCTTCTC
**IL-6**	GAGGATACCACTCCCAACAGACC	AAGTGCATCATCGTTGTTCATACA
**LGR5**	GGGCGTTAAGTCCACTGTGT	CGAACACCTGCGTGAATATG
**NFkB**	AAGACGGTGCTGGAGTCTGT	AGACGGTGCTGGAGTCTGT
**TGF-β1**	CTCCCGTGGCTTCTAGTGC	GCCTTAGTTTGGACAGGATCTG
**TNF-α**	AGCCCATGTTGTAGCAAACC	AGGAGGTTGACTTTCTCCTGGT

Table S2: Sequences of the forward (F) and reverse (R) primers used in the RT-PCR reaction for the amplification of the target gene.
